# Interphase tuning for stronger and tougher composites

**DOI:** 10.1038/srep26305

**Published:** 2016-05-27

**Authors:** Konstantin Livanov, Lin Yang, Asaf Nissenbaum, H. Daniel Wagner

**Affiliations:** 1 Department of Materials & Interfaces, Weizmann Institute of Science Rehovot 76100, Israel

## Abstract

The development of composite materials that are simultaneously strong and tough is one of the most active topics of current material science. Observations of biological structural materials show that adequate introduction of reinforcements and interfaces, or interphases, at different scales usually improves toughness, without reduction in strength. The prospect of interphase properties tuning may lead to further increases in material toughness. Here we use evaporation-driven self-assembly (EDSA) to deposit a thin network of multi-wall carbon nanotubes on ceramic surfaces, thereby generating an interphase reinforcing layer in a multiscale laminated ceramic composite. Both strength and toughness are improved by up to 90%, while keeping the overall volume fraction of nanotubes in a composite below 0.012%, making it a most effective toughening and reinforcement technique.

Realities of the modern world demand that engineering materials simultaneously possess high stiffness, strength and impact toughness, which is not a trivial task^1^. Typically, stiff and strong materials such as ceramics are brittle, whereas tough materials, for example rubber, are soft and weak. On an Ashby plot^2^ this translates into an inverse correlation between strength and toughness^3^. Such problematic behavior, however, is much less pronounced in natural composites like nacre^4^, bone^5^, turtle shell^6^ or sponge spicule^7^, where a number of complex reinforcing mechanisms (including crack bridging, crack deflection and geometric/structural intricacy) provide resistance to fracture propagation and impact toughness^8^. The possibility of applying similar mechanisms in synthetic materials is an important – albeit difficult – target of modern materials science and engineering. Recently a number of research groups have indeed succeeded in preparing remarkably tough composites by adapting such mechanisms to inherently brittle materials^9^,^10^,^11^.

Key parameters affecting the impact toughness of composites are the quality and strength of interfaces (quasi-two-dimensional boundaries between phases) and interphases (three-dimensional regions between phases)^12^, as schematically illustrated in Fig. 1a. If mechanically weak (in the broad sense), these regions may both limit the material stiffness and divert cracks, leading to an increase in overall toughness^13^. In fact, it is possible to engineer the interfaces/interphases so as to “direct” cracks and toughen the material. Interfacial failure can therefore be viewed as a “rate determining step”, as in a chemical reaction. A notable example of this approach is the work by Barthelat *et al*.^14^, where the controlled introduction of microdefects (and thus the creation of new interfaces) into monolithic glass structures has resulted in more than a hundredfold increase in structural toughness.

Another potentially effective method to improve material toughness is through the introduction of reinforcing elements, such as nanoparticles or carbon nanotubes (CNTs)^15^. Either in their single-walled (SWNT) or multi-walled (MWNT) versions, carbon nanotubes seem especially suitable as reinforcing elements due to their exceptional stiffness^16^, tensile strength^17^ and elongation to failure^18^. SWNTs and MWNTs have been successfully used to improve the mechanical characteristics of polymers^19^, ceramics^20^ and composite materials^21^. In most cases, however, CNTs are introduced as bulk reinforcement in the matrix of fiber-based composites, leaving the fiber-matrix interfaces relatively unaffected. As far as we know, however, only little research has been performed to selectively reinforce interphase regions of materials with CNTs. One advantage of such selective reinforcement is the very low volume fraction of CNTs needed to achieve an effect, hopefully positive, compared to bulk reinforcement.

Following our previous work, we concentrated on planar laminated structures made of ceramic layers separated by thin interlayers of polymeric adhesives^9^. This is inspired by the layered structure of sponge spicules, as explained in our previous work. Here, however, a thin network of carbon nanotubes was created on the surface of the ceramic layers. Note that this is contrast with the traditional method that consists in dispersing CNTs in the polymer matrix prior to composite preparation. We used evaporation-driven self-assembly (EDSA)^22^, a technique which generates a few nanotube-thick coating on a smooth substrate^23^ (Fig. 1b). In essence, the EDSA technique is based on the self-assembly process that occurs to dispersed particles or nanotubes upon evaporation of a solvent^24^. The dispersed nanotubes arrange themselves on the walls of a container or on any other vertically aligned flat surface. This is commonly known as the “coffee ring effect”, which has been well-studied^25^. In principle, the thickness and uniformity of the EDSA-produced coating can be precisely controlled. Moreover, EDSA does not require complicated equipment or expensive materials, and can be easily replicated. Additional information on the EDSA process can be found in the SI and references therein.

To create the nanotube network, MWNTs were dispersed in deionized water with the help of an ionic surfactant. Cleaned (using sonication in DI water, then in ethanol and acetone) alumina (Al_2_O_3_) slides were placed into specially designed tanks, and immersed in the dispersion. The tanks were left to dry for 72 hours at room conditions under a constant air flow. The solvent gradually evaporated, leaving a thin coating on the alumina surface (Fig. 1b). After complete evaporation, the slides coated with nanotubes were rinsed in ethanol to remove any excess of nanotubes and surfactant, and then analyzed by thermogravimetry (TGA) and electron microscopy. TGA under oxygen resulted (Fig. 1c,d) in a small but not insignificant weight loss (0.012%) at the temperature corresponding to MWNT evaporation^26^.

Figures 1e,f show an optical photograph of an alumina slide and a SEM image of the network, revealing its morphology at different scales. As seen, the EDSA-induced coating consists of interconnected bundles of CNTs, termed here “CNT network” or CNT-n. Darker and lighter domains or stripes on the specimen (Fig. 1f) correspond to higher and lower MWNT concentrations on the surface; as seen, no region is completely devoid of nanotubes, (for additional high magnification images, refer also to Figure S1 in the SI). The stripes arise due to the nature of the EDSA process^22^. The SEM image on Fig. 1 was obtained without gold-sputtering of the alumina substrate; thus the CNT-n film is conductive enough to prevent electron microscope beam damage to the sample. The CNT-n thickness was estimated from TGA measurements data (Fig. 1c,d). Assuming the MWNT density to be 1.8 g/cm^3.^^27^, the average thickness of the CNT-n coating is 64 nm, thus a few multi-walled nanotubes only, which corresponds well with the SEM observations (the diameter of single nanotubes, based on SEM, is 12–15 nm, see Figure S3 in the SI).

Spin-coating was then used to prepare sandwich-type composites with ceramic layers and polymer interlayers, similarly to what was performed in our previous work^9^. Two alumina slides with CNT-n coatings and with spin-coated adhesive interlayers of polyvinyl alcohol (PVA) were held together at 120 °C under a constant pressure to evaporate the excess solvent and reduce the amount of air bubbles, resulting in an Al_2_O_3_-PVA-Al_2_O_3_ sandwich-type composite with a ~2–3 µm thick PVA interlayer (Fig. 2a). Control samples without CNT-n were prepared exactly in the same way. The process was repeated to achieve the desired number of layers (2, 3, 4 and 6). For illustration, Fig. 2a shows a 2-layer composite. After preparation, the composites were carefully cut to the desired dimensions (Figure S5a in the SI) using a diamond saw. Based on the TGA results and the calculated average CNT-n thickness, the average volume fraction of CNT-n in a sandwich composite was found to be 0.025%.

All composites phases and interphases were carefully studied by electron microscopy (Fig. 2a1–a4). Inset a1 shows an alumina layer prior to the EDSA process; a2 – alumina layer coated with CNT-n after EDSA process (similar to Fig. 1f); a3 – PVA polymer wetting the CNT-n after spin-coating; and a4 – CNT-n completely wetted by and transferred to the polymer interphase. The hardness of the polymer-alumina interphase, with and without CNT-n, was measured by nanoindentation. The specimens that contained nanotube networks were found to be almost 100% harder than those with no CNT-n (0.26 ± 0.02 GPa and 0.14 ± 0.04 GPa, respectively).

3-point bending tests were performed to determine the strength and work of impact fracture (W_f_) of the composites. The crosshead speed during all the measurements was relatively high, 3 m/s, to simulate impact. Representative load-displacement plots are shown in Fig. 2b–e for 2, 3, 4 and 6-layer composites, respectively. In all cases the CNT-n reinforced specimens failed at higher loads and had larger area under the load-displacement plot.

Toughness/work of impact fracture and strength were calculated for all specimens as described in the Methods section. The results are summarized in Table 1. The column for “1-layered” specimens relates to single alumina layers with and without CNT-n coating, without polymer interlayer, and is included for comparison purposes. In all cases, reinforced specimens are both stronger and tougher than their plain counterparts. The observed decrease in strength (both in plain and CNT-reinforced composites) with the number of layers is likely due to poor interfacial stress transfer of the polymer interlayers – the more interlayers, the less stress is transferred to the next alumina layers, due to imperfect adhesion between polymer and alumina^28^. The appearance of air bubbles and matrix defects during composite preparation enhances this effect even further^29^. Contrasting with this, the work of impact fracture increases with the number of layers, in agreement with both literature and our previous work^30^,^31^,^9^, with the exception of the 6-layered specimens. We assume that the latter exhibit lower mechanical properties, both in terms of strength and W_f_, due to less effective heating due to their thickness, and as a result, formation of larger amounts of air bubbles during specimen preparation. In case of “1 layer” specimens, i.e. CNT-n coated alumina layers with no polymer interlayer, the difference between reinforced and plain results is negligible, as expected. In other words, in the absence of a polymer interlayer, CNT-n coating plays no reinforcing role.

Our interpretation of the reinforcement mechanism, based on the SEM analysis of the fractured specimens, is illustrated in Fig. 3. Extensive observations, including from previous works^9^, appear to confirm that layer delamination is the main crack deflection mechanism in this type of composites, arising from the shear stress induced by the 3-point loading configuration^30^. A delaminating crack, in plain and CNT-n reinforced specimens, is schematically shown in Fig. 3a,b, respectively. As delamination proceeds, the alumina layer, having much higher stiffness, remains unaffected, but the polymer interphase undergoes significant plastic deformation, starting at the tip of delamination crack. In a reinforced specimen, the interphase deformation region (“ID” on Fig. 3a,b) contains a high concentration of carbon nanotubes. Delaminated PVA surface, shown on Fig. 3c,d, contains numerous surface deformations, indicating that PVA has filled the gaps between the surface grains of the ceramic, providing strong cohesive forces for the laminates. In Fig. 3d, almost all CNTs are embedded in the polymer matrix and do not expose their ends out, indicating that the superior mechanical performance of the reinforced composite does not result from occasional nanotube bridging between the ceramic and the PVB, but likely from the plastic deformation of the CNT-reinforced interphase. As the crack grows, the plastic zone around the crack tip moves forward, and the deformed polymer behind the crack tip unloads. This plastic loading and unloading around the crack tip, as well as post-debonding friction between the polymer and alumina^32^ leads to energy dissipation, which contributes to fracture toughness^33^.

As the applied load increases, the plastic strain of the polymer at the interface has to grow to a certain degree to cause the delamination crack growth. We can assume that the interphase with CNT-n embedded into the polymer matrix (Fig. 2a4, refer also to Figures S7 and S8a in the SI) would yield higher interphase shear modulus than the pristine polymer. This is supported by both nanoindentation hardness measurements and published works^34^. Given the larger interphase shear modulus, during the development of the plastic zone at the crack tip, the plastic strain of the reinforced polymer interphase should require more energy than that of plain polymer, leading to a larger plastic zone. This difference between plain and reinforced plastic zones is schematically illustrated on Fig. 3a,b (“PZ” vs. “RPZ”). The dissipated energy generated by delamination crack growth at the reinforced interphase is thus larger than that for the plain one.

Figure 3e,f, show side-view SEM images of the interphase deformation zone of the reinforced composite, illustrating the many distortions and deformations of the interphase, caused by delamination. Such distortions are common for soft matrix composite materials, and are known as “shear hackles”^35^. As the delaminating crack progresses, the shear hackles are subjected to tensile forces, tear and pull out (similar to fibers, pulled out of the matrix in an oblique manner^36^). In the case of a reinforced specimen, the shear hackles contain high concentrations of carbon nanotubes (Fig. 3f), that ensure higher strain energy release rates of the processes mentioned above. In addition, some top-view SEM images of the reinforced interface show longitudinal cracks, with CNT-n branches bridging (Figures S8a,b in the SI). All these factors contribute to the toughening of the CNT-n reinforced specimens, which is confirmed here to be a complex multiscale, multi-mechanism process^32^. Note that the failure is likely a mixture of Mode I and Mode II failure, however from the shapes of the “hackles” and from observations during the fracture experiments Mode I appears to be more prevalent.

As discussed previously, the yielding strength of the laminates is governed by the efficiency of stress transfer^29^ and the interfacial shear strength^15^,^37^,^38^ of the composite. We speculate here, based on observed data and published works^33^, that the CNT-n reinforced interlayers have higher interfacial shear strength and thus provide better stress transfer than plain ones. Since the strength of the composites decreases with the number of polymer interlayers, it can be said that the CNT-n reinforcement somewhat compensates for this effect.

To demonstrate the universality and applicability of the interphase reinforcement mechanism, we prepared two-layer sandwich composites with glass layers (instead of alumina) and PVB interlayers (instead of PVA), mimicking standard two-ply glass widely used in car windows and other applications^39^. Common microscope slides were used as glass substrates. Figure 4a is an optical photograph of such a slide, with a CNT-n coating arranged in a characteristic “stripe” pattern. Figure 4b shows a SEM micrograph of the CNT-n coated glass slide. The control and reinforced substrates were spin-coated with a PVB solution, and 2-layer composites were prepared in the same manner as before. 3–point bending specimens were shaped into desired dimensions using a diamond saw. The average volume fraction of CNT-n in the glass-PVB sandwich composite was found to be 0.012%.

Figure 4c shows typical load-displacement traces for the plain and reinforced composites, which again shows a significant positive effect of the CNT-n interphase reinforcement on the fracture process. It thus appears that the reinforcement mechanism is not dependent on the type of substrate or polymer matrix, and is likely to be active in other layered materials based on soft polymer matrices. Figure 4d shows a comparison between the flexural strength and work of impact fracture of plain and reinforced composites. In both cases the advantage of the reinforced samples is apparent.

Another essential parameter of 2-ply glass-PVB composites is the optical transmittance, for example for car windows applications. Figure 4e shows the optical transmittance measurements of plain and reinforced samples, underlining the advantage of the CNT-n coating which allows the use of very small amounts of nanotubes. The resulting ~80% transmittance of the reinforced specimen (dark blue line in Fig. 4e) is not only higher than other CNT-reinforced 2-ply glasses with 0.5 and 1.5% CNT (~40% and ~20% transmittance, respectively, red lines in Fig. 4e)^40^, but is also higher than the 70% minimum transmittance required for real-life applications in car windows^41^ (grey line in Fig. 4e).

Table 2 demonstrates the effectiveness of interphase reinforcement by comparing strength and toughness reinforcement efficiency for this and selected literature works. Stress reinforcement efficiency (η_σ_) was calculated by dividing the composite strength increase after reinforcement (Δσ) by the total CNT volume fraction in the composite:


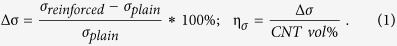


The resulting dimensionless number represents the efficiency of strength reinforcement in composites. Toughness reinforcement efficiency (η_R_) was calculated by the same manner:


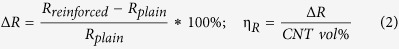


where R is one of the toughness parameters: impact toughness, fracture toughness or (as in this work) work of fracture. If the amount of carbon nanotubes in a composite was given in the literature in terms of weight rather than volume fraction, a conversion was made using the CNT density value of 1.8 g/cm^3^ 27. Literature examples include bulk reinforcement of polymers prepared via solvent^19^,^37^,^42^,^43^,^44^ and melt^45^ nanotube dispersions, CNT grafting on fibers in fiber-polymer composites^21^, bulk reinforcement of ceramic composites via CNT insertion prior to sintering^20^,^46^ and CNT-reinforced laminates^40^,^43^,^47^,^48^. As can be convincingly seen from the data in Table 2, interphase reinforcement is more than an order of magnitude more efficient than other known reinforcement pathways.

To summarize, we present here a new approach designed to reinforce (both in terms of strength and toughness) layered composites using carbon nanotubes. This approach utilizes the premise that interfacial/interphasial failure is a key early step in the failure of layered composites. The method is based upon preparing the composite material in such a way that significant concentrations of carbon nanotube reinforcement are present only in the interphase area (thus, not in the bulk, Fig. 1a), which significantly improves the mechanical properties of the interphase. In this way, very small amounts of carbon nanotubes are enough to produce a significant effect. The reinforcement effect arises from a multiscale combined effect of well-known reinforcement mechanisms (delamination, plastic zone growth, crack bridging, and oblique CNT pull-out). It is shown here that this interphase reinforcement approach is general as it can be applied to various substrates. It also can be scaled up or down from the centimeter-size substrates shown in this manuscript, making it suitable for various production volumes. As such, it has extensive appeal for potential applications.

## Methods

### The EDSA process

MWNT dispersion was prepared as follows. CVD-grown MWNTs (50 mg) were put into 100 ml of DI water and sonicated for 20 minute. Then, 0.5 g. of sodium dodecyl sulphate (SDS) was added to the dispersion, which was sonicated for a further 2 hours. Resulting dispersion was stable in ambient conditions for several weeks. Al_2_O_3_ 0.25 mm thick plates (99.6% pure, as-fired, unpolished, purchased from Valley Design Corp., Shirley, MA) were cut using a diamond saw to 30 mm × 10 mm substrate slides. The slides were sonicated in DI water, ethanol and acetone and then put into a specially designed EDSA tank (see Figure S5b in the SI). MWNT dispersion was filtered through cotton wool to remove large aggregates, and then added to the tank until the substrates were completely covered by the liquid. The tanks were left in ambient conditions for 72 hours until all the water evaporated. CNT-n coated substrates were gently rinsed with ethanol to remove excess SDS and dried under an air stream. The same procedure was repeated for the glass substrates.

### Composite preparation

Polyvinyl alcohol (PVA) (99.7% purity, 78000 Mw, hydrolyzed; Polysciences, Inc., Taipei, Taiwan) was gradually dissolved in heated DI water until the concentration reached 17 wt%. A Laurell WS650 spin-coater (North Wales, PA) was used at 5000 rpm for 45 s on both CNT-n treated and untreated Al_2_O_3_ substrates. The final thickness of a single spin-coated polymer layer was ~1–1.5 μm as measured by gravimetric analysis and by focused ion beam FIB spectroscopy. After spin-coating, two alumina slides with PVA coatings were held together under 1.5 kg pressure and heated to 120 °C for 2 min to evaporate the excess solvent and reduce the amount of air bubbles, resulting in an alumina–polymer–alumina sandwich composite with a ~2–3 μm polymer interlayer in-between. The process was repeated to reach the required layered composite thickness (i.e. 2, 3, 4 and 6 layers).

Glass-PVB composites were prepared in the same way. Polyvinyl butyrate (PVB) was gradually dissolved in heated dimethyl formamide (DMF) until the concentration reached 20 wt%. After spin-coating, two glass slides with PVB coatings were held together under 1.5 kg pressure and heated to 160 °C for 2 min. The rest of the procedure was exactly the same.

### 3-point bending

Alumina and glass composites of various thicknesses were carefully cut using a diamond saw, resulting in final lateral dimensions of 30 mm × 5 mm. 3-point bending tests were performed on a Bose ElectroForce 3200 UTM instrument, with crosshead speed of 3 m/s, to simulate impact. All specimens were pre-loaded at 2 N prior to fracture. At least seven specimens of each type were tested.

Flexural strength and work of impact fracture were calculated from the load-displacement plots. Strength (*σ*) was obtained as follows:


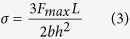


where *L* is the support span, *b* is the specimen width, *h* is the specimen height and *F*_*max*_ is the maximum force^9^.

The work of impact fracture (*W*_*f*_) was calculated as follows:


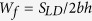


where *S*_*LD*_ is the total area under the load-deflection curve^9^.

### SEM analysis

**Figure 1:**
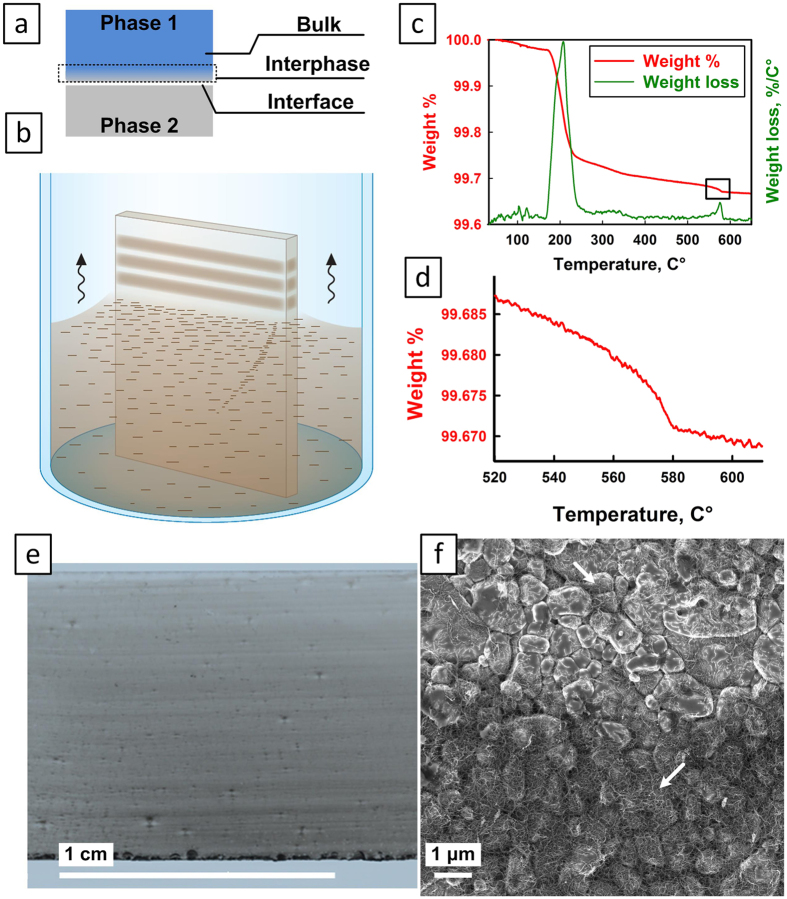
EDSA process. (**a**) Schematic representation of 3D interphase. (**b**) Schematic illustration of the EDSA process. (**c–d**) TGA weight % (red) and weight loss (green) plots of the CNT-n coated Al_2_O_3_ substrate and an enlarged portion of the red graph. (**e**) A photo of CNT-n coated Al_2_O_3_ substrate. (**f**) SEM image of CNT-n coated Al_2_O_3_ substrate, showing the boundary between a darker and lighter region. White arrows point to CNT bundles. Scale bars: (**e**) 1 cm; (**f**) 1 μm.

High-resolution scanning electron microscopy (HRSEM) pictures were taken using SUPRA-55 VP Zeiss and ULTRA-55 Zeiss (Oberkochen, Germany) instruments using an In-Lens detector. Images were collected at acceleration voltage of 3 kV and working distance of 4–5 mm. In some cases, the SEM specimens were used as-is (Figs 1 and S1 in the SI); In other cases to prevent sample charging, the samples were sputtered with gold–palladium alloy prior to SEM imaging, using an Edwards (Sanborn, NY) S150 sputter coater.

## Additional Information

**How to cite this article**: Livanov, K. *et al*. Interphase tuning for stronger and tougher composites. *Sci. Rep.* **6**, 26305; doi: 10.1038/srep26305 (2016).

## Supplementary Material

Supplementary Information

## Figures and Tables

**Figure 2:**
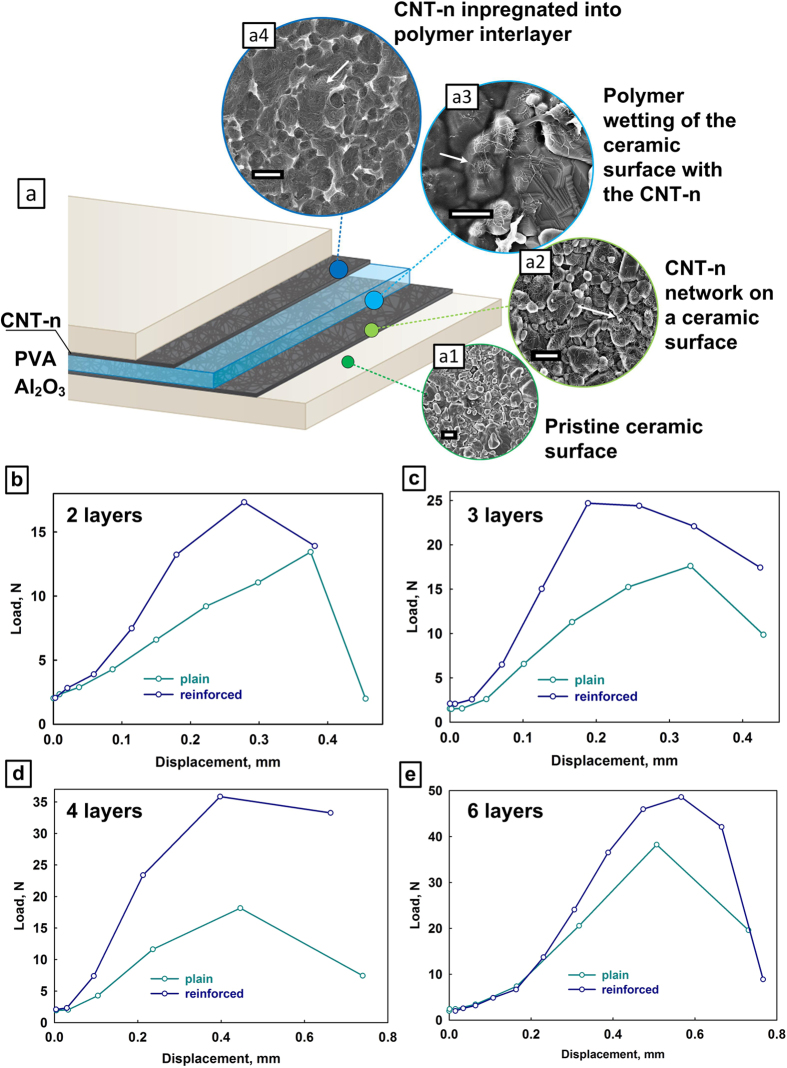
Multiscale layered composite structure and mechanical properties. (**a**) Schematic illustration of a 2-layer CNT-n reinforced composite with short descriptions of each layer and preparation stage. (a1–a4) SEM images of various phases of the composite. Full-scale a1–a4 images can be found in the SI. The arrows point to CNT bundles. (**b–e**) Representative load-displacement plots of the plain and reinforced composites of 2, 3, 4 and 6 layers. Scale bars: (a1–a4) 1 μm.

**Figure 3:**
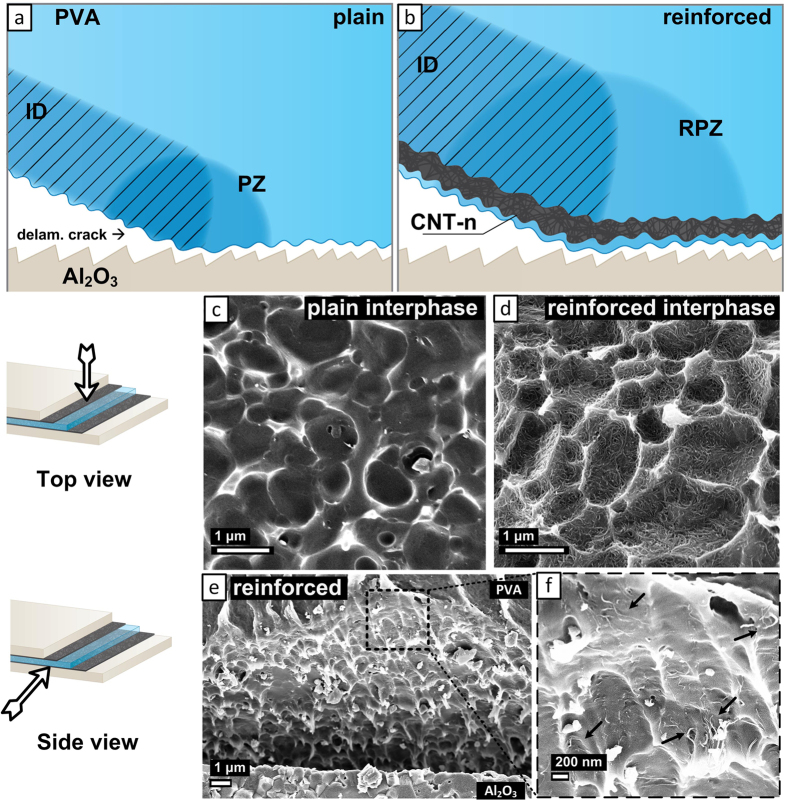
Reinforcement mechanism. (**a,b**) Schematic illustrations of the delaminating crack propagation in a plain (**a**) and reinforced (**b**) specimen. ID stands for “interphase deformation”, PZ for “plastic zone”, and RPZ for “reinforced plastic zone”. (**c,d**) Top-view SEM images of the plain (**c**) and reinforced interphase. (**e**) Side-view SEM image of the reinforced interphase. (**f**) Zoom-in of (**e**); black arrows show CNT fibers protruding from the PVA matrix. Scale bars: (**c–e**) 1 μm; (**f**) 200 nm. Additional SEM images of the interphase can be found in the SI.

**Figure 4:**
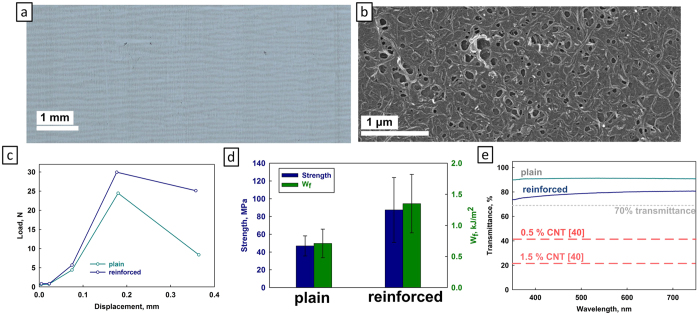
Glass-PVB composites. (**a**) Optical photograph of a glass microscopy slide, showing the CNT-n coating. (**b**) SEM image of the CNT-n coated glass slide. (**c**) Representative load-displacement plot of the plain and reinforced glass-PVB composites. (**d**) Comparison between the plain and reinforced composites’ strength (blue) and work of impact fracture (green). (**e**) Transmission plots for plain and reinforced glass composites compared to literature analogues (red lines, adapted from [40]) and industrial standard^41^. Scale bars: (**a**) 1mm; (**b**) 1 μm.

**Table 1:** Mechanical properties of layered composites.

**Number of layers**	**1**^a^	**2**	**3**	**4**	**6**
Strength (plain) [MPa]	439 ± 125	205 ± 63	152 ± 32	124 ± 22	96 ± 17
**Strength (reinforced) [MPa]**	**483** ± **119**	**239** ± **51**	**179** ± **53**	**186** ± **29**	**131** ± **16**
W_f_ (plain) [kJ/m^2^]	0.66 ± 0.29	0.99 ± 0.46	1.29 ± 0.31	2.36 ± 0.42	1.99^b^ ± 0.27
**W**_**f**_ **(reinforced) [kJ/m**^**2**^]	**0.72** ± **0.17**	**1.21** ± **0.21**	**2.07** ± **1.46**	**4.02** ± **0.76**	**2.59**^b^ ± **0.30**

^a^Single alumina layers without polymer interlayer. This column is included for comparison purposes.

^b^Lower numbers possibly due to ineffective heating during sample preparation.

**Table 2:** Comparison of strength and toughness reinforcement efficiencies for current and selected literature works.

**Specimen**	**Reinforcement type**	**CNT vol%**^a^	**Δσ**^b^**, [%]**	**Reinforcement efficiency (η**_**σ**_)^c^	**ΔR**^d^**, [%]**	**Reinforcement efficiency (η**_**R**_)^e^	**Reference**
Al_2_O_3_-PVA (4L)	Interphase	0.025%	50%	2000	70%	2800	This work
Glass-PVB	Interphase	0.012%	89%	7415	90%	7500	This work
PC	Solvent dispersion	5%	32%	6.4	–	–	42
Epoxy	Solvent dispersion	0.3%	–	–	17%	57	43
Epoxy	Solvent dispersion	0.5%	25%	50	46%	92	44
PMMA	Melt dispersion	1%	–	–	170%	170	45
Carbon fiber-Epoxy	Fiber grafting	0.5%	–	–	40%	80	21
Al_2_O_3_	Ceramic	10%	–	–	194%	19.4	20
Al_2_O_3_	Ceramic	10%	–	–	9%	0.9	46
Glass-PVB	Laminate	1.5%	30%	20	341%	227	40
Preform-Epoxy	Laminate	0.3%	–	–	48%	160	43
SiC fabric-Epoxy	Laminate	2%	240%	120	348%	174	47
PEI/PAA	Layer-by-layer	50%	2400%	48	–	–	48

^a^Whenever applicable, calculated from weight fraction (wt%) using the CNT density of 1.8 g/cm^3 ^27.

^b^Calculated by: Δσ = (σ_reinforced_ − σ_plain_)/σ_plain_ * 100%, where σ is the reported composite yield strength.

^c^Calculated by: η_σ_ = Δσ/CNT vol%.

^d^Calculated by: ΔR = (R_reinforced_ − R_plain_)/R_plain_ * 100%, where R is the reported composite toughness, fracture toughness or work of fracture.

^e^Calculated by: η_R_ = ΔR/CNT vol%.
