# Supplementary material for: Interphase tuning for stronger and tougher composites
Source: Sci Rep. 2016 May 27;6:26305. doi: 10.1038/srep26305 (PMC4882545; doi:10.1038/srep26305)
Supplement: Supplementary Information [file srep26305-s1.pdf]

Supplementary Information

for

Interphase tuning for stronger and tougher composites

K. Livanov, L. Yang, A. Nissenbaum, H.D. Wagner
Department of Materials & Interfaces, Weizmann Institute of Science
Rehovot 76100, Israel

1. SEM images of CNT-n on alumina surface after the EDSA process

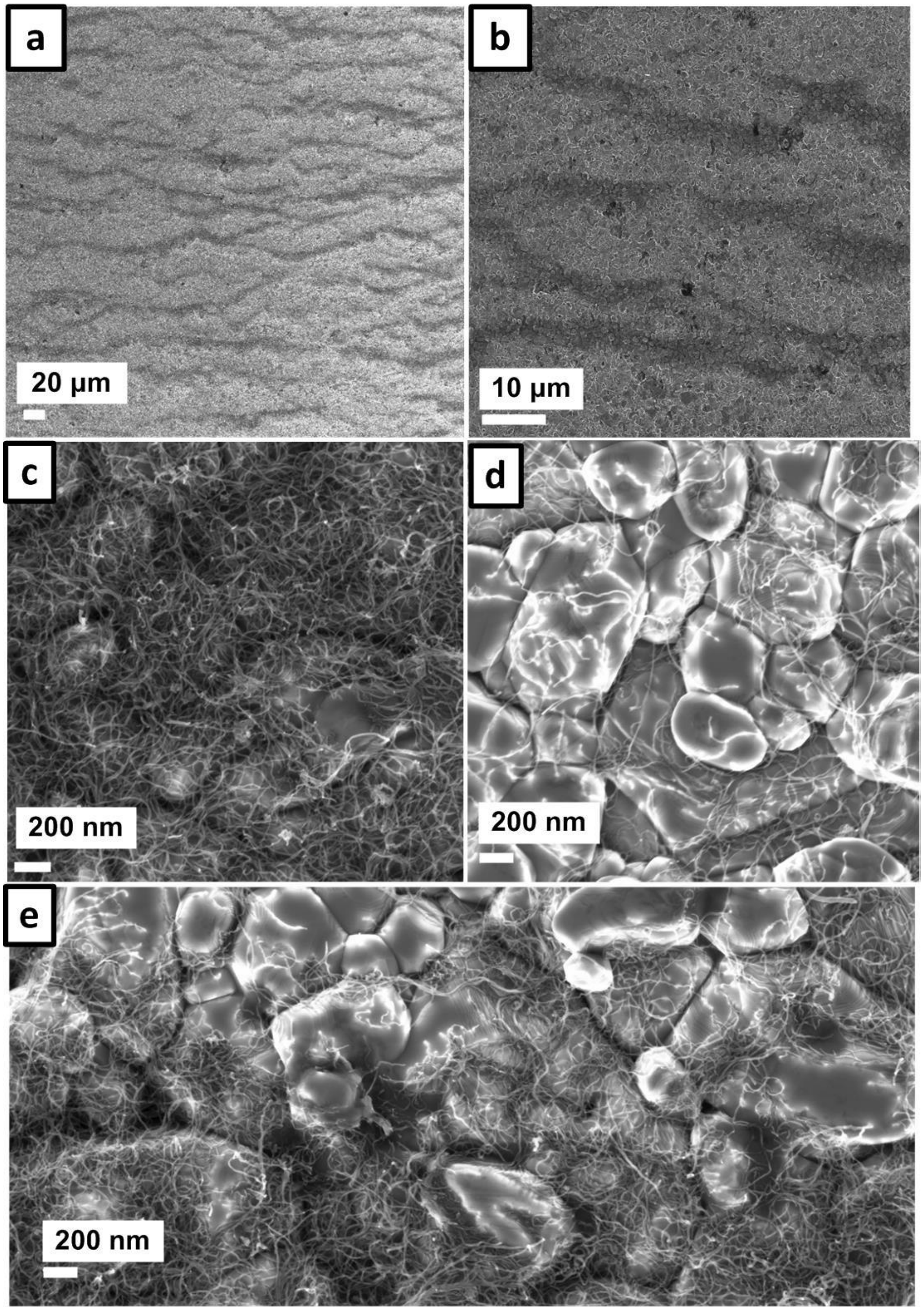

Figure S1: SEM images of CNT-n dispersed on a Al_2O_3 substrate at different manifications. a) and b) are general low-magnification images. c) is a magnified image of a darker region. d) is a magnified image of a lighter region, clearly showing the nanotube coating. e) is a magnified image of a transition between the lighter (top) and darker (bottom) regions. Scale bars: a) 20 µm, b) 10 µm, c-e) 200 nm.

2. EDSA process

EDSA process is a result of a complex balance between the liquid's surface tension, the friction force of the contact line and evaporation. The simplest model often described in the literature is a single drop of liquid with dispersed particles ("coffee droplet") on a flat surface [S1].

When a drop of coffee is left on a solid surface to evaporate, it leaves a dense agglomeration of coffee particles at the periphery of the droplet, while much less particles are deposited inside the droplet. This "coffee ring" phenomenon is a well-known example of the general form of EDSA (Figure S2a).

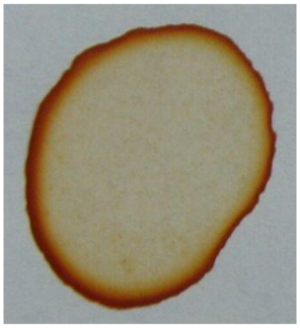

Figure S2a: Dense deposition of coffee particles at the droplet periphery.

When a droplet spreads on a non-ideal surface (i.e. surface that has some degree of roughness) it (1) forms a cape due to the surface tension and (2) the contact line (the triple liquid-solid-gas interface) is getting pinned to the surface. Further, we assume that evaporation always takes place and is constant in an open system and in stable proper conditions (temperature/pressure). When a droplet is drying on a surface, evaporation reduces the height of the droplet at any point on the droplet surface. In a case of an ideal surface, where no contact line pinning takes place, the droplet would shrink to maintain its spherical shape, retained by the surface tension. In this ideal case, the system has a single contact angle. However, on non-ideal surfaces the contact line is pinned and the droplet cannot shrink to compensate on the liquid loss. In this case, the compensation is done by a flow of liquid from the center of the droplet to the periphery [S1],[S2]. Figure 2 displays this mechanism.

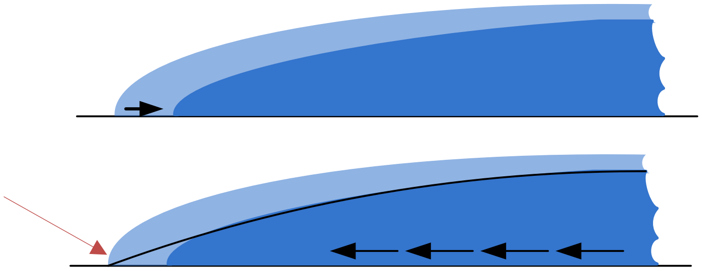

Figure S2b: (top) No pinning of contact line. The droplet shrinks as evaporation occurs. (bottom) The contact line is pinned. The black curve is the change in the droplet profile due to the contact line pinning and the evaporation. The red arrow points at a portion of solution that has been evaporated but needs to be compensates by the flow of liquid from inside [S1].

When liquid flows from inside the droplet to the periphery, it will carry any particles that are suspended in it. Since evaporation of liquid continues as long as there is liquid available, a constant flow occurs simultaneously as well, thus taking a major portion of the particles to the periphery. Another important phenomenon associated with EDSA is the stick-slip movement of the contact line. The stick-slip movement of the contact line can be visually seen as discrete concentric rings with a gradient of deposit concentration. This phenomenon was thoroughly investigated in the literature [S3, S4, S5]. On an ideal surface, there is no pinning of contact line and therefore, the system maintains a single contact line, governed by the surface-tension of the three phases. But on a non-ideal surface, where the pinning force is stronger than the surface tension, the contact angle changes, and the shape of the droplet changes with it. At a certain point, the system is so far from equilibrium that the tension forces of the droplet overcome the pinning force. In that case, the contact line detaches from its initial position and recedes to a new point where the system is again in equilibrium (i.e. the contact angle is θi). The process then will continue, and a dense agglomeration of particle will form at the new location of the contact line.

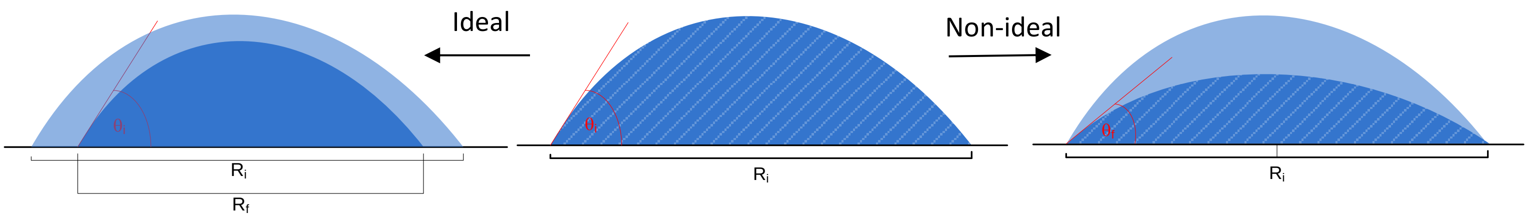

Figure S2c: Two evaporation-induced mechanisms for ideal (left figure) and non-ideal(right figure) surfaces.

This simplified explanation assumes that while the droplet evaporates only one thermodynamic parameter is changing while the other is constant. Experimentally it is usually not the case, and on non-ideal surfaces, both contact angle and contact line will vary together, which leads to less discrete, but rather continuous coating of the surface.

According to the literature [S3],[S4],[S5] this droplet model can be transferred to the case of a flat vertical substrate in the evaporating liquid. The function of the droplet contact line in this case is transferred to the meniscus contact line. The EDSA coating mechanism therefore results from competition between the meniscus surface tension, the coatings surface tension, and the friction force at the contact line.

3. SEM images of pristine carbon nanotubes and pristine clean alumina

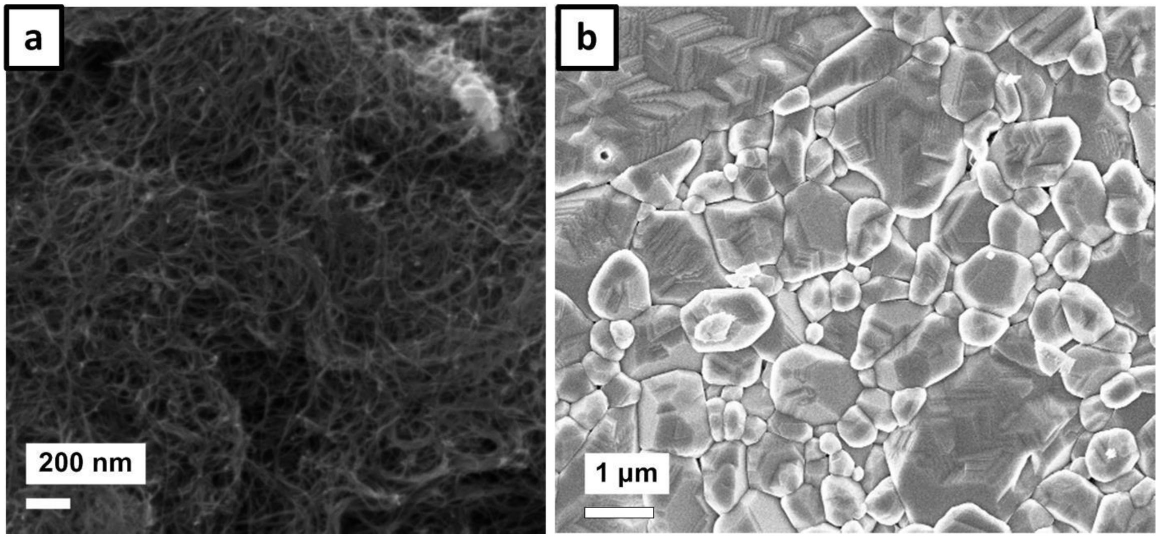

Figure S3: SEM images of a) pristine multi-wall carbon nanotubes and b) pristine cleaned Al_2O_3 substrates used in this work. Scale bars: a) 200 nm, b) 1 µm.

4. Enlarged versions of Figure 2 a1-a4.

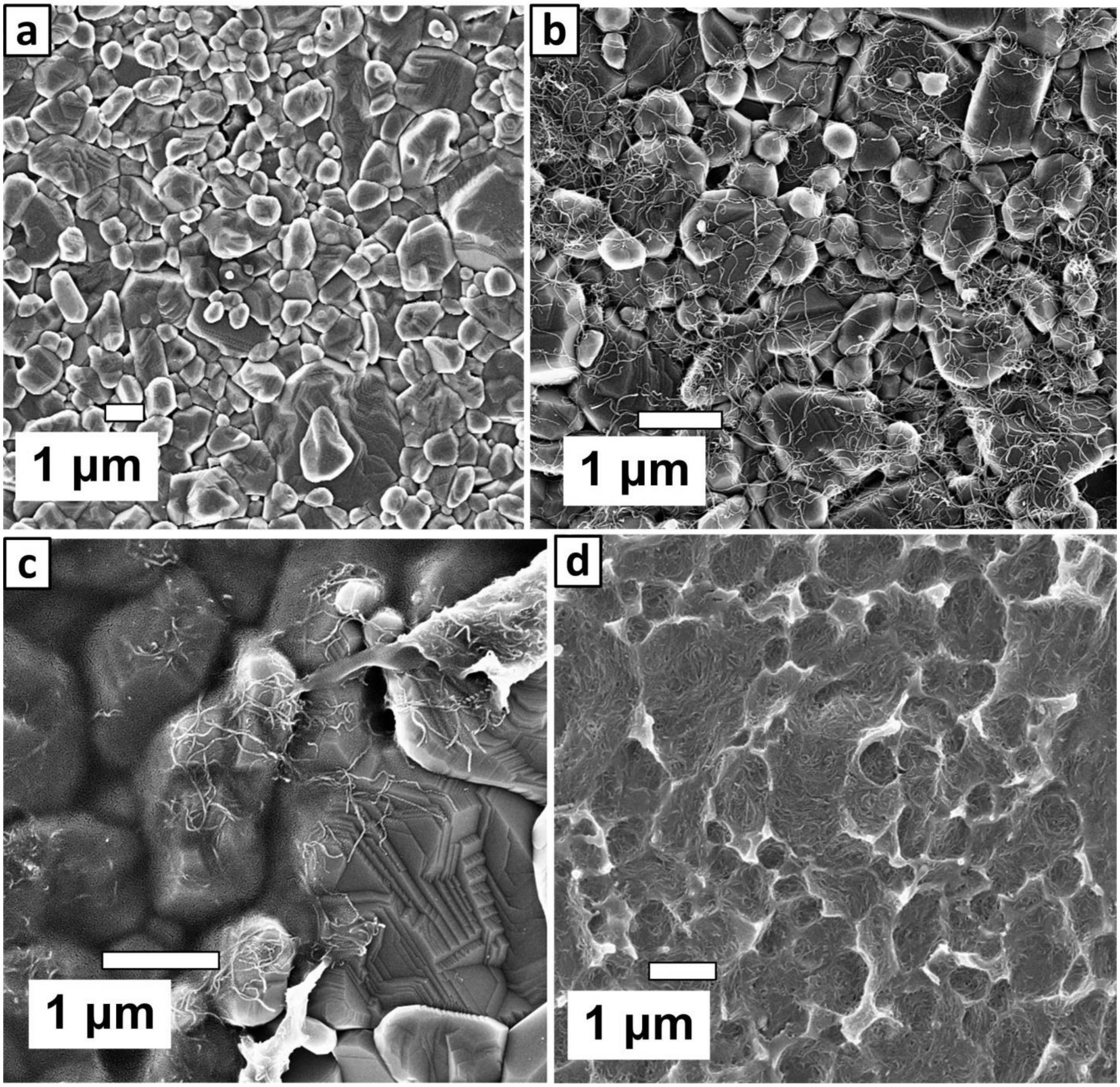

Figure S4: a-d) Enlarged and rectangle versions of Figures 2a1-a4 respectively. Scale bars: 1 µm.

5. Dimensions of bending samples

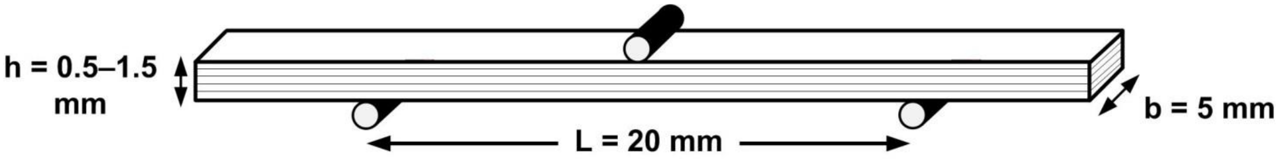

Figure S5: Dimensions of an Al_2O_3-PVA layered composite specimen for 3-point bending. The specimen thickness varied according to the number of layers: from 0.5 mm for 2 layers to 1.5 mm for 6 layers; each layer added 0.25 mm.

6. Nanoindentation data

Nanoindentation was performed on a peeled-off polymer interphase (such as seen on Figure 3c,d in the main article body or on Figures S7 and S8a in this Supporting Information) with and without carbon nanotubes (plain and reinforced samples). The nanoindenter used was an Agilent XP nanoindenter, with a 10 micron spherical diamond tip. The continuous stiffness (CSM) method was applied to get continual reading of modulus with depth. Indentations were made to depth of 1 micron.

Hardness as measured by nanoindentation for the plain PVA interphase: 0.14 ± 0.04 GPa

Hardness as measured by nanoindentation for the CNT-n reinforced PVA interphase: 0.26 ± 0.02 GPa

It is important to note that the measurement was challenging due to high surface roughness and adhesion.

7. CNT-n wetted by the PVA

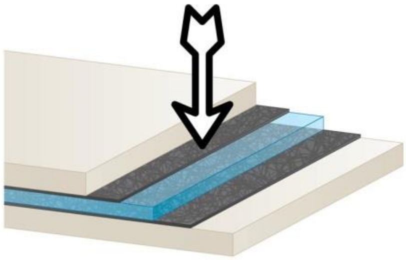

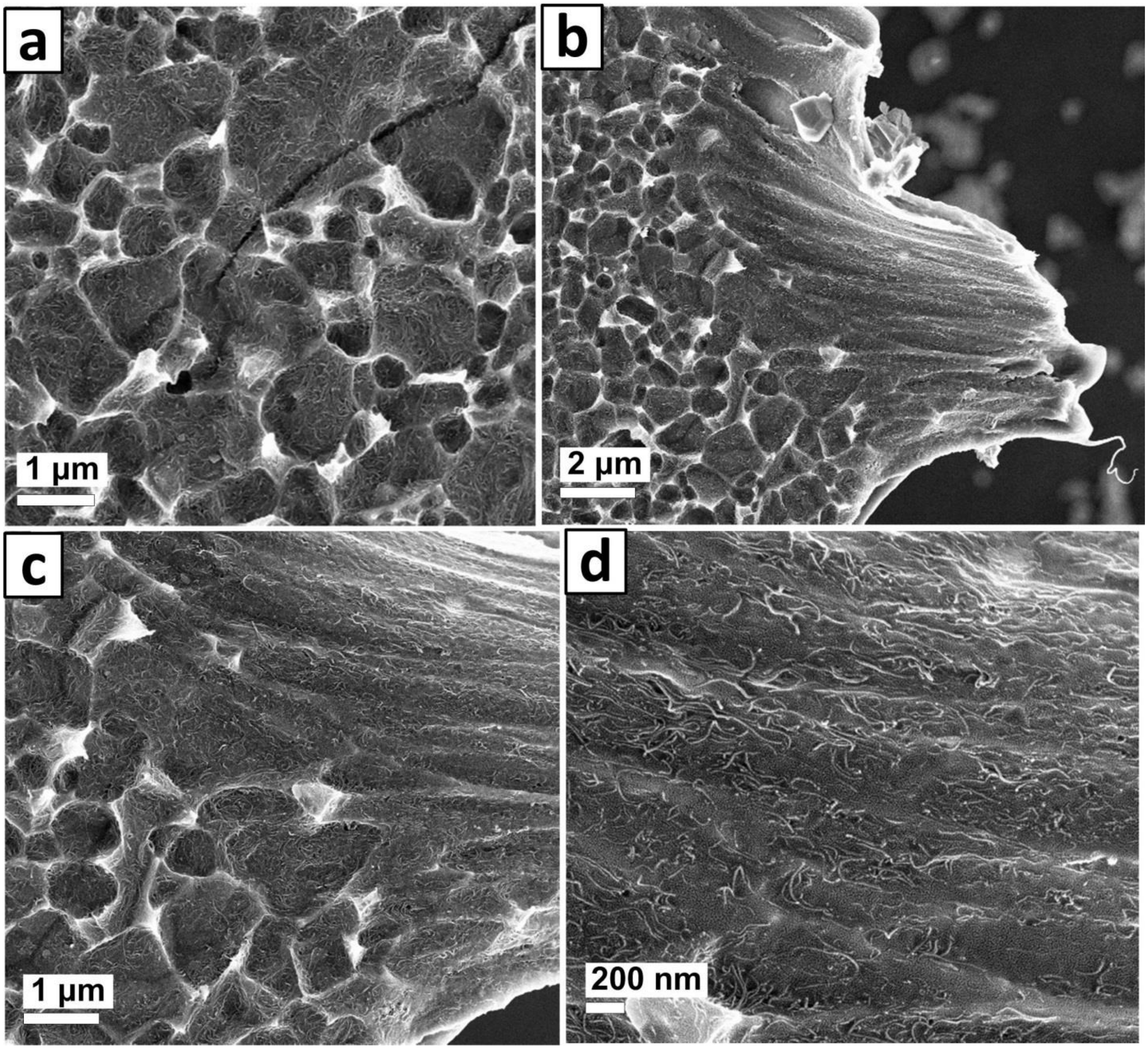

Figure S7: SEM images of CNT-n reinforced interphase (top-view) (additional images to Figures 2a4 and 3d in the main body), showing several types of defects occurring at the interface: a) a long crack and b) plastic deformation of the polymer. c) and d) are zoom-ins of b). Scale bars: a), c) 1 µm, b) 2 µm, d) 200 nm.

8. Bridging of CNT-n reinforced PVA surfaces

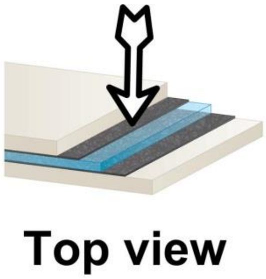

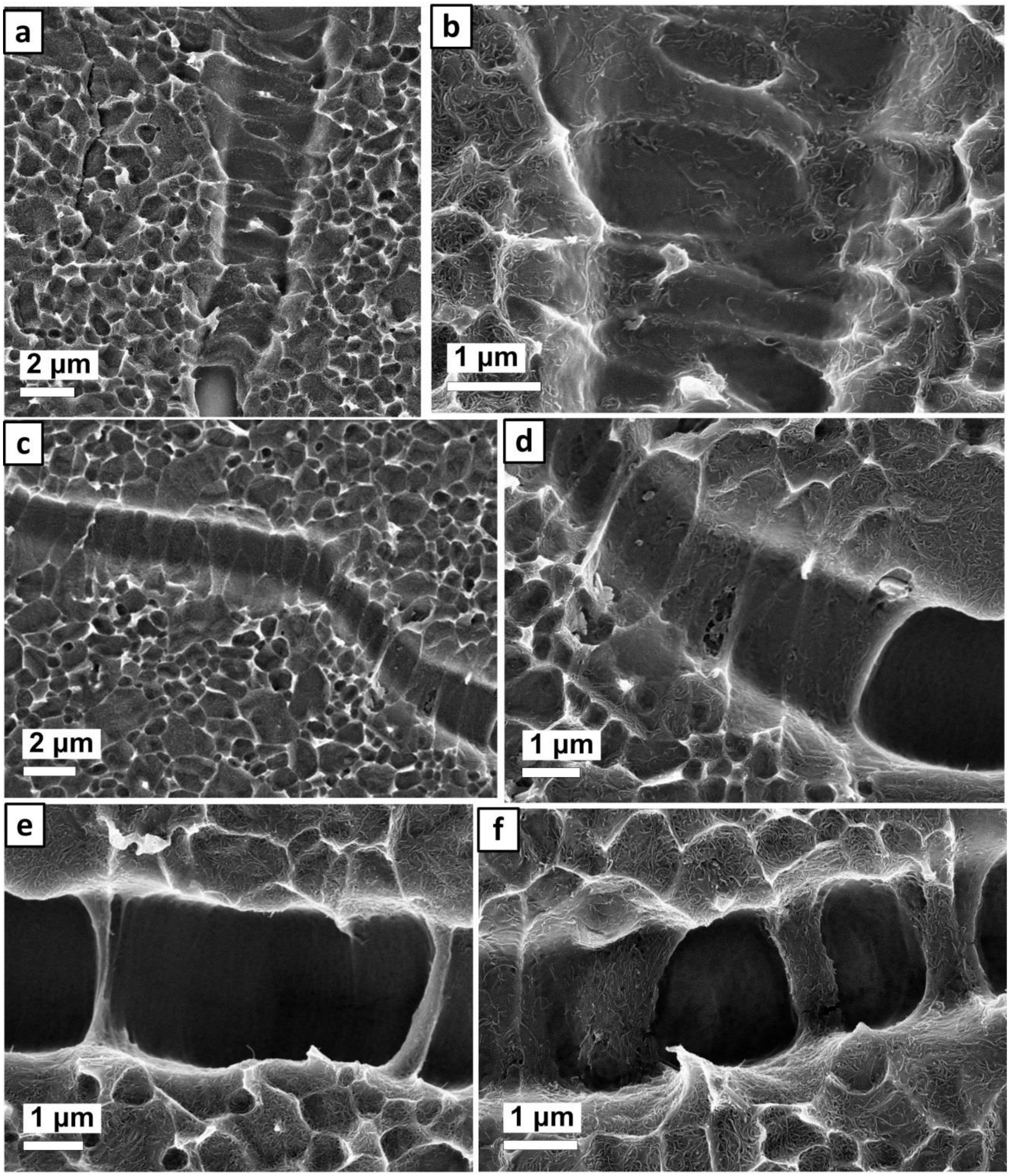

Figure S8a: SEM images of CNT-n reinforced interphase (top-view) (additional images to Figures 2a4 and 3d in the main body), showing CNT-n bridging mechanism. b) and d), e), f) are zoom-ins of a) and c) respectively. Scale bars: a), c) 2 µm; b), d-f), 1 µm.

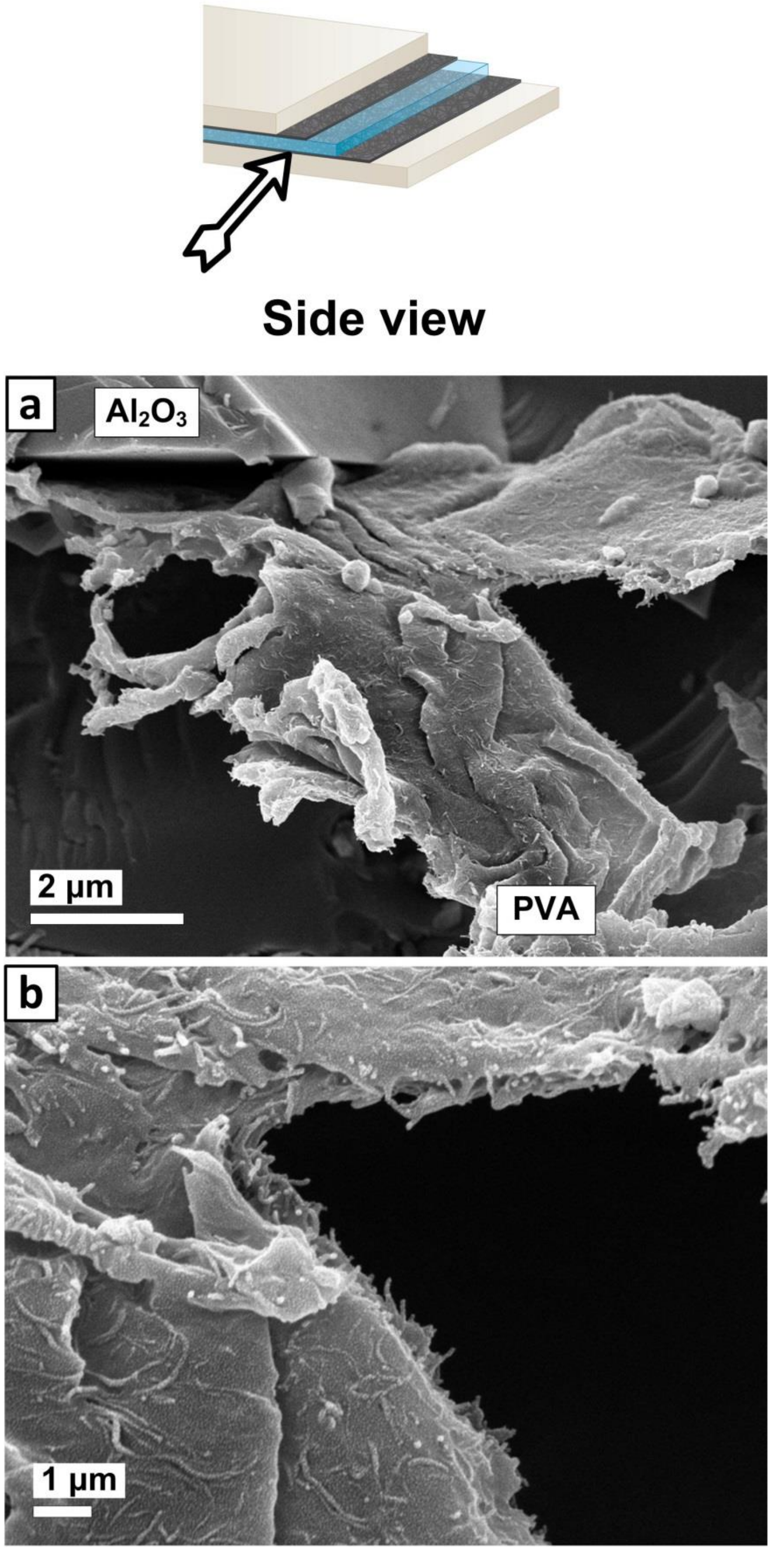
Side view
a
Al$_2$O$_3$
2 μm
PVA
b
1 μm

Figure S8b: SEM images of CNT-n reinforced interphase (side-view) (additional images to Figures 3e,f in the main body), showing CNT-n bridging and nanotube pull-out toughening mechanisms. b) is a zoom-ins of a). Scale bars: a) 2 μm; b) 1 μm.

9. Materials and Methods

UV-Vis measurements were taken with Cary 300 Bio UV-Visible spectrophotometer with air as a baseline.

TA Q-series SDT Q600 Thermal Gravimetric Analyzer was used in all TGA measurements. Alumina crucibles were used.

10. References

[S1] R. D. Deegan, O. Bakajin, T. F. Dupont, G. Huber, S. R. Nagel, T. A. Witten, "Capillary flow as the cause of ring stains from dried liquid drops", *Nature*, 389, **1997**, 827

[S3] E. Bormashenko, Y. Bormashenko, R. Pogreb, O. Stanevsky, G. Whyman, "Droplet behavior on flat and textured surfaces: Co-occurrence of Deegan outward flow with Marangoni solute instability", *J Colloid Interface Sci*, 306, **2007**, 128–132

[S3] E. Adachi, A. S. Dimitrov, K. Nagayama, "Stripe Patterns Formed on a Glass Surface during Droplet Evaporation", *Langmuir*,11, **1995**, 1057-1060

[S4] Y. Mino, S. Watanabe, M. T. Miyahara, "*In Situ* Observation of Meniscus Shape Deformation with Colloidal Stripe Pattern Formation in Convective Self-Assembly", *Langmuir*, 14, **2015**, 4121-8

[S5] A. S. Dimitrov, K. Nagayama, "Continuous Convective Assembling of Fine Particles into Two-Dimensional Arrays on Solid Surfaces", *Langmuir*, 12, **1996**, 1303-1311
